# Development of a sample preparation protocol for fast fluorine screening of sealing materials—Considering potential PFAS regulations

**DOI:** 10.1007/s00216-026-06575-2

**Published:** 2026-06-12

**Authors:** Sebastian Kampf, Ronya Mona Wallis, Lennart Gehrenkemper, Björn Meermann

**Affiliations:** 1 Federal Institute for Materials Research and Testing (BAM), Division 1.1 – Inorganic Trace Analysis (ITALab), 12489 Berlin, Germany; 2 German Environment Agency (UBA), FG II 2.5 “Laboratory for Water Analytics”, 12099 Berlin, Germany

**Keywords:** Elastomers, PFAS, Fluorine, HR-CS-GFMAS

## Abstract

Elastomers commonly used as sealing materials are highly networked rubbery polymers. They may contain different pollutants such as fluorinated compounds like per- and polyfluoroalkyl substances (PFAS), for example used as additives to enhance the performance of the elastomer under a variety of chemical and mechanical stresses. As of now, no reliable recycling strategy for elastomers is available and they are consequentially disposed of by incineration or in landfills. Due to the potential wash out of additives or other pollutants into landfill leachates, elastomers present a possible source of continuous environmental pollution. A processing protocol for elastomers is required as a basis to enable the reliable determination of extractable fluorine: Here, we describe a quick and robust sample preparation protocol to determine extractable fluorine in various sealing materials. The optimization was aimed for time efficiency and robustness of results; sample preparation was conducted for fluorine sum parameter analysis via high resolution-continuum source-graphite furnace molecular absorption spectrometry (HR-CS-GFMAS). Samples were first roughly shredded to 2 mm and then further milled and sieved to defined particle size fractions. Different size fractions were tested and the extraction of the size fraction between 125 and 250 µm was found to be the best trade-off between extraction efficiency, time efficiency and sample loss. Subsequent methanolic or aqueous extraction and HR-CS-GFMAS analysis of various polymer samples revealed quantifiable extractable fluorine content for most samples. In the future, the sample processing method developed could make a valuable contribution as a standard operating procedure (SOP) for a time-efficient sample preparation for subsequent extraction and screening of sealing materials in prospect of the strict regulation on PFAS proposed by the European Chemicals Agency (ECHA).

**Graphical Abstract:**

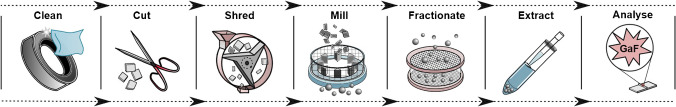

**Supplementary Information:**

The online version contains supplementary material available at 10.1007/s00216-026-06575-2.

## Introduction

Environmental pollution is ever increasing through industrialization [1], though regulations have been enacted and iteratively updated to limit the release of pollutants into the environment. One group of pollutants raising public concern is per- and polyfluoroalkyl substances (PFAS), anthropogenic chemicals containing at least one fully fluorinated methyl or perfluorinated methylene group [2, 3]. Due to their favourable properties, such as oil and water repellence, surface-activity as well as heat and chemical resistance, PFAS are used in a wide range of products and industrial processes [4]. The excessive use has led to the global spread of PFAS throughout rivers, oceans, soil and air [5]. Environmental entry paths range from industrial manufacturing sites, to the usage of products by customers to their disposal as waste [6]. The environmental fate of PFAS is determined by the specific physicochemical properties of a particular PFAS substance and the environmental medium [5], with the trend of long-chain carboxylic and sulfonic acid PFAS to be more persistent in soil, sediments and biota compared to short-chain PFAS [5, 7–10]. PFAS have frequently been detected in both marine and terrestrial biota [9, 11–17]. Moreover, studies indicate a plethora of adverse health effects on humans [6]. These include the disruption of hormone signalling such as the endocrine system and the hypothalamic-pituitary-gonadal axis [18–20], cardiovascular [21] and metabolic disorders [22] as well as cancer [23–25]. The problem of PFAS exposure and their effects on human health and the environment is further complicated by the large and increasing diversity of PFAS compounds, as it is difficult to ((eco-)toxicologically, and analytically) assess the multitude of (emerging) substances with different properties [26].

The adverse effects of PFAS contamination have given reasons for concern and efforts are made to regulate the use and release of PFAS in response: The Stockholm Convention, a worldwide agreement to eradicate or at least reduce the use of persistent organic pollutants (POPs) has included the terminal PFAS perfluorooctanoic acid (PFOA), perfluorohexanesulfonic acid (PFHxS), perfluorooctanesulfonic acid (PFOS), and their respective salts as well as any long-chain perfluorinated carboxylic acids with a carbon chain length between nine and 21 C-atoms [27]. Further, since 2007 the regulation on the registration, evaluation, authorization, and restriction of chemicals (REACH) has taken effect in the European Union. Under this legislation chemical compounds exceeding certain production volumes or importation quantities need to be registered before being manufactured, imported or placed on the EU market and are thus evaluated in the perspective of environmental and human protection. There are currently several exceptions from REACH and although monomers need to be registered, the associated polymers, additives (necessary to preserve the stability of the polymer), impurities and intermediate products during polymerization are exempt from the registration obligation [28]. This exemption represents a blind spot in the REACH regulation especially considering that the polymer industry in, e.g., the Nordic countries, is the highest-grossing industry using PFAS during product manufacturing [4]. In addition, residues of fluorine-containing catalysts used during polymerization [29] may remain in the polymer. As polymer additives, they may enhance the resistance to temperature, chemical and mechanical stresses [4, 29, 30]. During polymer production PFAS may be used as mould release or foaming agent potentially contaminating the final product [4, 30, 31]. Additionally, monomers can be (per-)fluorinated themselves resulting in highly fluorinated polymers such as fluoroelastomers (e.g., fluorocarbon-based fluoroelastomer (FKM)) and polytetrafluoroethylene (PTFE) [30]. Elastomers, sometimes referred to as rubbers [30], are a subgroup of polymers and exhibit elastic properties [32] due to their networked structure [29]. Owed to their chemical resistance, flexibility and swelling behaviour when in contact with solvents, elastomers are frequently used as sealing materials [29]. However, since no reliable recycling strategies for elastomers are currently available without compromising the quality of the resulting polymer, they are often disposed of either by combustion or in landfills [33]. It was frequently shown that landfill leachates can be contaminated with PFAS [34–38] and that the leachate may contaminate the immediate environment as well as groundwater reservoirs [39]. Even before disposal, sealing materials can degrade when exposed to harsh environments [40] which may also lead to leaching of additives [41] or other pollutants. Furthermore, these issues are complicated by the fact that the exact composition of polymers and elastomers is often unknown, as are the additives used [42, 43].

The ECHA is currently discussing a more strict regulation of the production and usage of over 10,000 PFAS [44]. Time periods for phase outs are discussed for a plethora of PFAS applications including the use in sealing materials, adhesives and polymers for varying years after the enactment of the legislation [45]. Especially for applications where PFAS are still seen as indispensable such as their use in proton exchange membranes or in sealing applications within industrial sites [44], it is imperative to reliably screen for both PFAS content and their leaching potential. In prospect of a group regulation of PFAS with a broad PFAS definition, these insights may advance the development of materials sustainable and safe by design and therefore reduce or even prevent the unintentional release of PFAS into the environment before eventually being phased out entirely.

Thus, this study aims to provide a robust and quick standard operation procedure (SOP) for the fast processing of various sealing materials as a basis for the screening of extractable fluorine. The SOP is comprised of cutting, shredding, and sieving the samples to defined size fractions to allow for the subsequent extraction and determination of fluorine—in light of the plethora of PFAS compounds—by fluorine sum parameter analysis via high resolution-continuum source-graphite furnace molecular absorption spectrometry (HR-CS-GFMAS) [46–48]. In addition, trials for the extractability of fluorine into water were performed as a potential indication for additive wash out from polymers at the size of microplastics [49] which naturally are generated through particle abrasion [50] and during the filling and storage of landfills [51]. The development of an SOP to process various polymer blends will in future enable the development of additional sample extraction and preparation protocols to rapidly screen in terms of safe and sustainable by-design materials as well as allow for verification of compliance to potential regulatory demands. Further measurements (e.g., (non-)target) or sample preparation (e.g., solid-phase extraction (SPE)) may be implemented to expand the measurements beyond extractable fluorine and allow for the differentiation between inorganic fluorine and organically bound fluorine such as PFAS.

## Materials and methods

### Reagents

Perfluorooctanoic acid (PFOA) was used as calibration standard (98% purity, *J&K Scientific GmbH*, Pforzheim, Germany). Methanol (*Chemsolute*® HPLC-Grade) and acetic acid were obtained from *Th. Geyer GmbH & Co KG*, (Renningen, Germany). Calcium nitrate (Ca = 1 g/L), magnesium nitrate (Mg = 1 g/L), zirconium nitrate (Zr = 1 g/L) and palladium nitrate (Pd = 1 g/L) solutions; all *Certipure®* grade purchased from Merck KGaA*,* Darmstadt, Germany; were used as modifier for HR-CS-GFMAS measurement and diluted according to Metzger, Ley [46]. Ultra-pure water (UPW) was generated using a *Milli-Q® Millipore, Q-Guard® 2* system (Merck KGaA*,* Darmstadt, Germany).

### Sample acquisition

Samples consisting of the following polymers were obtained: Nitrile-butadiene rubber (NBR), ethylene-propylene-diene-monomer rubber (EPDM), styrene-butadiene-rubber (SBR), polyurethane rubber (PUR), isoprene isobutylene rubber (IIR), polytetrafluoroethylene (PTFE), chloroprene rubber (CR) and fluorocarbon-based fluoroelastomer (FKM). Samples were provided by sealing material producers and processors near Berlin (Germany) (Table 1; SI: Figs. 1–12).

**Table 1:** Samples investigated during this study as well as their texture

Polymer	Sample name	Texture
Ethylene-propylene-diene	EPDM1	Solid
Ethylene-propylene-diene	EPDM2	Porous
Styrene-butadiene rubber	SBR1	Solid
Styrene-butadiene rubber	SBR2	Solid
Chloroprene rubber	CR1	Porous
Chloroprene rubber	CR2	Solid
Nitrile-butadiene rubber	NBR1	Solid
Nitrile-butadiene rubber	NBR2	Solid
Isoprene-isobutylene rubber	IIR	Solid
Polyurethane rubber	PUR	Solid
Polytetrafluoroethylene	PTFE	Solid
Fluoroelastomer	FKM	Solid

### Sample processing

Samples were cleaned with dry, lint-free laboratory wipes. Before shredding, samples were cut to smaller pieces (maximum side length 1 cm). Samples were weighed, submerged in liquid nitrogen for 1 min and transferred to an *SM 200* cutting mill (*Retsch*®, Haan, Germany) equipped with an 8 mm bottom sieve. The machine was set to 2611 rcf. The shredded samples were collected, and the cooling and shredding was repeated using a 2 mm bottom sieve. To prevent melting or thermal degradation during upcoming milling steps the shredded samples were cooled for 3 min using liquid nitrogen. Cooled samples were milled using a *ZM 200* centrifugal mill (*Retsch*®, Haan, Germany) equipped with a 500 µm ring sieve and rotation set to 10,831 rcf. The cooling and milling were carried out twice with identical procedures. For a preliminary test, the samples obtained after shredding with the cutting mill were fractionated with an *AS 200* vibratory sieve shaker (*Retsch*®, Haan, Germany) equipped with two sieves with mesh sizes of 1000 µm and 500 µm. For the investigation of smaller particle size fractions, the obtained sample after additional usage of the centrifugal mill was fractioned with two sieves with mesh sizes of 250 µm and 125 µm. Applying this procedure the following fractions were obtained for each sample: 1: 250–500 µm; 2: 125–250 µm; 3: < 125 µm. If necessary, samples were cooled with liquid nitrogen before sieving. However, cooling of all samples before sieving is recommended to reduce electrostatic interactions between sample particles.

To prevent cross contamination, each sample preparation procedure was followed by a cleaning procedure: All mills and sieves were cleaned with a vacuum cleaner and compressed air. All movable parts were rinsed with UPW and ethanol. The moveable parts were dried with compressed air and laboratory wipes. Precleaned sieves were sonicated in UPW at RT for 10 min, rinsed with UPW and dried in a drying oven overnight.

### Methanolic and aqueous fluorine extraction

Extraction of samples was done according to a modified protocol of Simon, Gehrenkemper [52]. Samples were weighed to 1 g ± 10% into 15 mL polypropylene (PP) centrifuge tubes. 5 mL of methanol acidified with 0.5% acetic acid (*v*/*v*) were added to the centrifuge tube resulting in a liquid to solid ratio of 5:1 (*v*/*w*). Samples were shaken vigorously for 30 s, sonicated for 5 min at RT and subsequently centrifuged for 10 min at 4500 rcf. The supernatant was collected in a 50 mL PP centrifuge tube. The contact time of the extractant was approximately 20 min. Overall, the extraction step was repeated four times, and the supernatants were combined. The solvent was evaporated under a gentle stream of nitrogen. After drying, samples were stored at −18 °C in the dark until further use. Each sample was extracted as triplicate.

The aqueous extraction procedure was identical to the methanolic extraction but was performed with UPW as extraction solvent. Instead of evaporation under a stream of nitrogen, the collected and combined supernatant after four consecutive extraction steps was passed through filter paper (*black ribbon*, cellulose, 589, Schleicher & Schüll GmbH, Dassel, Germany). Filtrates were collected in new 50 mL PP centrifuge tubes. The water was removed by freeze drying (*Alpha 2–4 LASC*, Martin Christ Gefriertrocknungsanlagen GmbH, Osterode am Harz, Germany). Before freeze drying, the centrifuge tubes were sealed with *Parafilm®* (*Parafilm® M PM-992*, Merck KGaA, Darmstadt, Germany). To allow for the evaporation of the solvent, the *Parafilm®* was perforated several times using a clean syringe needle. Freeze drying took place at −85 °C under vacuum (2·10^3^ mbar) and was continued until the pressure and temperature were stable. Any powdery residue on the inside of the *Parafilm®* was reunited with the sample. After drying, samples were stored at −18 °C in the dark until further use. Each sample was extracted as duplicate.

Before measurement, samples were reconstituted in 1 mL methanol:UPW (1:1 (*v*/*v*)), vigorously shaken and sonicated at RT for 10 min. For both extractions, procedural blanks were prepared and handled identically to the sample extractions. Blank values were subsequently subtracted from the calculated fluorine content of the samples.

### High resolution-continuum source-graphite furnace molecular absorption spectrometry

Determination of extractable total fluorine was conducted by means of a *ContrAA 800* (Analytik Jena AG, Jena, Germany). The data were processed using the corresponding software *Aspect CS 2.2.2.0* (Analytik Jena AG, Jena, Germany). The oven was equipped with a graphite furnace with PIN-platform (Analytik Jena AG, Jena, Germany). A modified protocol by Metzger, Ley [46] was followed for the temperature program and sample injection protocols. Before first use, the PIN-platform was coated with Zr solution (35 µL, 1 g/L) and conditioned with Ca solution (25 µL, 20 mg/L) and modifier mix (0.1% (*v*/*v*) Pd matrix modifier, 0.05% (*v*/*v*) Mg matrix modifier, 20 mg/L Zr standard) following the temperature protocol provided by Metzger, Ley [46]. Samples were injected according to SI: Table 1 following the temperature program in SI: Table 2. Ga was used as molecule forming agent for the measurement of GaF at the strongest molecular absorption line at 211.248 nm [53]. Measurements were performed in instrumental quadruplets. Sample extracts were diluted with methanol:UPW at 1:1 (*v*/*v*) to fit within the external calibration if necessary.

Measurements were calibrated against PFOA. A linear regression was obtained for the following fluorine concentrations: 5, 10, 25, 50, 75, 100, 150, 250 µg/L. The matrix of the standard solution was matched to the matrix of the samples measured (methanol:UPW at 1:1 (*v*/*v*)). The instrumental and methodological LOD and LOQ were on average 5.5 and 18.2 µg/L, respectively.

## Results and discussion

### Investigation of the effect of particle size and size distribution on fluorine extraction

Either directly used as catalyst, monomers, as additives or as contamination during production, fluorine may be present (as organic or inorganic compounds) within elastomers often used as sealing materials [4, 29, 54, 55]. Hence, a sample processing protocol was developed applicable to various sealing materials. As proof of concept, extractable fluorine (EF) was measured as sum parameter via HR-CS-GFMAS. The reproducibility of the determination of EF was examined to establish the optimal size fraction for subsequent extraction and analysis.

**Fig. 1:**
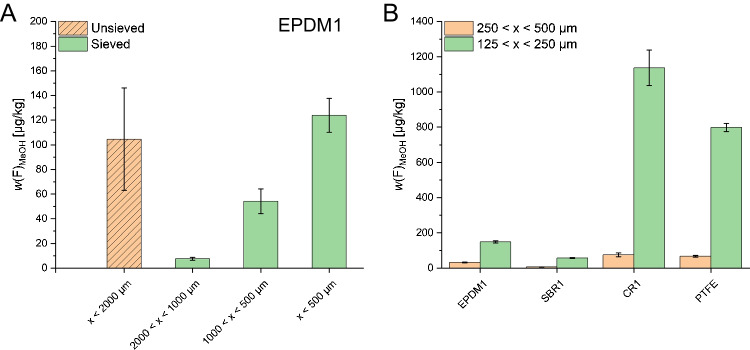
Determination of the extractable fluorine content depending on the size fraction via HR-CS-GFMAS. Samples were extracted with acidified methanol. **A** Preliminary test: Extractable fluorine determined in sample EPDM1 after initial shredding with a cutting mill and subsequent sieving to three size fractions (*n* = 3). **B** Extractable fluorine determined in EPDM, SBR, CR and PTFE samples shredded, milled and sieved to two size fractions between 500 and 125 µm (*n* = 3). Error bars represent the standard deviation

The influence of the particle size and particle size distribution on the extraction efficiency of fluorine was investigated using the modified extraction protocol by Simon, Gehrenkemper [52]. Although sieving the shredded sample EPDM1 (Fig. 1A) initially decreased the extraction efficiency measured via HR-CS-GFMAS, the reproducibility increased notably as demonstrated by the decreasing relative standard deviation (RSD) in correspondence to the decreasing particle size (Table 2).

**Table 2:** Fluorine mass fraction *w*(F)_MeOH_ [µg/kg], standard deviation (SD) and relative standard deviation (RSD) measured in EPDM1 at different size fractions via HR-CS-GFMAS. Samples were extracted in triplicates according to a modified protocol by Simon, Gehrenkemper [52]

Size fraction [µm]	*w*(F)_MeOH_ [µg/kg]	SD [µg/kg]	RSD [%]
x < 2000 (unsieved)	104.6	41.5	39.7
2000 < x < 1000	7.7	1.2	15.6
1000 < x < 500	54.1	10.0	18.6
x < 500	123.9	13.8	11.2

The highest fluorine extraction efficiency and smallest RSD were observed for the smallest size fraction tested; < 500 µm (Fig. 1A, B). To examine even smaller particle size fractions, a centrifugal mill was used. The correlation between particle size and extraction efficiency was verified using four samples of different polymers. Two defined particle size fractions (500–250 µm and 250–125 µm) were investigated for this purpose (Fig. 1B). For all samples, a higher extractable fluorine content was observed for the smaller particle fraction, with varying degrees of increased extraction efficiency. This is likely due to the reduced surface-to-volume ratio and possibly due to more efficient swelling of the polymer particles in the solvent [30].

Use of smaller size fractions may further increase the extraction efficiency. However, four aspects were considered when the optimal particle fraction was selected: (i) The obtained sample particles, which were smaller than 125 µm, could not be assigned to any defined size fraction for technical reasons. (ii) The yield was highest for the fraction between 125 µm and 250 µm and there was a considerable loss of sample during processing (SI: Table 3). (iii) To achieve enough sample below 125 µm to subsequently analyse in technical triplicates, additional shredding cycles would have to be performed, substantially increasing the processing time and raising the risk of sample degradation due to heat-up and loss during cutting and milling. (iv) With decreasing size fraction, the sample particles became successively more electrostatic, likely due to interactions between polymer fragments. The consequentially increased sample clumping complicates sample handling and presents a possible cause of inhomogeneities. Overall, the use of the size fraction between 125 and 250 µm was chosen as a reasonable trade-off between processing time, sample loss, and increased extraction efficiency.

**Fig. 2:**
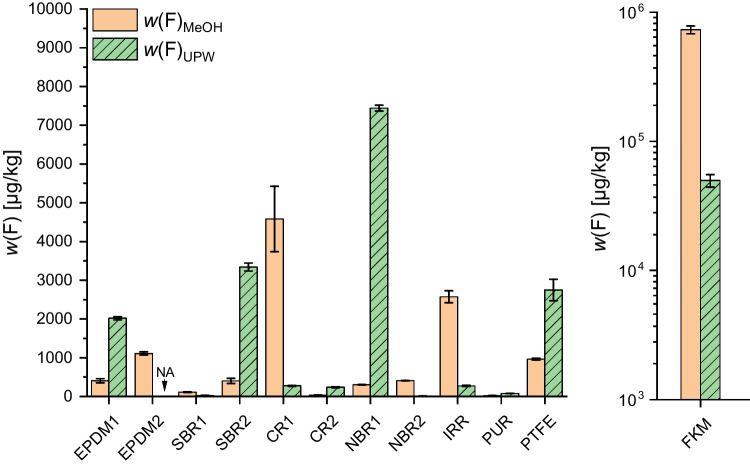
Mass fraction of extractable fluorine upon extraction with either acidified methanol (MeOH, n = 3) or ultra-pure water (UPW, *n* = 2) as extraction solvent from different processed sealing material samples determined via HR-CS-GFMAS. Error bars represent the standard deviation

The applicability of the sample processing procedure to different sealing materials was further investigated for 12 samples in total. EF was measured by HR-CS-GFMAS while the relative standard deviations between triplicates were used as a benchmark for reproducibility for the applied extraction procedure at the chosen size fraction. Additionally, fluorine was extracted using UPW to determine extractability of fluorine such as residues from fluorinated processing agents and additives from polymers at the size of microplastics (Fig. 2).

### Proof of concept for various sealing materials

In both extraction media, extractable fluorine was determinable with an average relative standard deviation of 7.8% and 7.7% between technical replicates in acidified methanol and UPW, respectively (Fig. 2). The reproducible results presented prove the sample processing protocol to be applicable to different sealing materials and different ranges of fluorine content. Although, the extraction protocol has been optimized for soil samples, the results suggest that it can be viewed as solid foundation for further optimization regarding polymeric matrices. Extractable fluorine was above the methodological LOQ of 18.2 µg/kg in all samples when extracted by methanol. For all samples except FKM, the determined fluorine concentration ranged from 26 µg/kg to 4600 µg/kg. Similarly for the aqueous extraction, all samples except NBR2 were above the methodological LOQ and ranged from 22 µg/kg to 7500 µg/kg, excluding FKM. In both the methanolic and aqueous extraction, FKM exhibited elevated fluorine contents of 7.3 × 10^5^ µg/kg and 4.9 × 10^4^ µg/kg, respectively.

FKM and PTFE have been chosen within this study specifically for being (per-)fluorinated polymers. Although depending on the exact composition, FKM is a polar, networked elastomer [29, 56], which may allow unreacted monomers and fluorinated additives to adsorb to the molecular structure. This may potentially have led to the elevated extractable fluorine content determined via HR-CS-GFMAS. Contrary to FKM, PTFE is a perfluorinated nonpolar polymer [29]. For PTFE, no ordinarily elevated extractable fluorine content was determined compared to the other samples examined, showcasing that fluorination of polymers does not necessarily correspond to an increased extraction of fluorine in either methanol or UPW. These findings are particularly insightful considering use-cases where fluorinated sealing materials cannot be avoided entirely.

The differences in extractable fluorine between methanolic and aqueous extraction are likely due to differences in analyte as well as solvent polarity and, in conjunction with polymer polarity, differences in liquid absorption and swelling, which enables the extraction of additives or residues [30]. In this study, 10 out of 11 samples showed that quantifiable amounts of fluorine were extracted into water under the applied conditions for the size fraction between 125 and 250 µm, raising concerns regarding the potential of leaching from polymer particles at the size of microplastics [49]. Assuming that landfill leachates are of varying compositions depending on the products disposed of and the amount of precipitation [57, 58], it can be expected that the potential wash out of fluorine and other pollutants from polymers depends on the combination of polymer properties and the exact environmental conditions. Considering the persistence of polymers to degradation in nature [59, 60] the continuous pollution through the release of additives or other pollutants such as PFAS poses a non-negligible risk and may contaminate the environment (and enter the food-chain) for years to come.

### Limitations

The potential SOP has been developed to quickly and reliably screen for extractable fluorine in polymeric samples with the focus on the sample preparation for subsequent extraction. Although the measurements are targeted towards PFAS sum-parameter analyses, no differentiation can be made between organically bound fluorine (OF) and inorganic fluorine (IF) for the presented results since measurement as GaF via HR-CS-GFMAS does not discriminate between fluorine-species. Given that HR-CS-GFMAS is known to exhibit fluorine species-specific responses it is important to consider the presence of IF within the sample—otherwise the results may be over- or underestimated depending on the external calibration used with IF such as NaF producing notably stronger responses compared to PFAS molecules [46, 61, 62]. As such, the measurement of OF is limited by the presence of IF. Sources of IF may be fluorine containing catalysts used for EPDM polymerization [54], co-initiators such as BF_3_ for IIR polymerization [55] or possibly unintentional contamination through tainted mineral fillers for varying polymers [63]. The presented SOP was developed to be expedited by additional sample preparation techniques such as solid phase extraction (SPE) to separate IF from the sample before measurement [64] to subsequently allow for the reliable screening of OF within a variety of sealing materials.

## Conclusion

We developed a potential standard operating procedure (SOP) for the processing of various sealing materials to allow for the subsequent determination of extractable fluorine content. The optimal size fraction for reproducible results was found to be the milled and sieved polymer samples between 125 µm and 250 µm, which represented an appropriate trade-off between processing time and sample loss. Consistent and reproducible results for extractable fluorine content were obtained in both methanolic and aqueous extractions. Processing of 12 different polymer samples according to the developed SOP with methanolic extraction and subsequent HR-CS-GFMAS measurements resulted in the determination of the extractable fluorine content above the LOQ for all samples and with small RSDs for technical triplicates. The successful extraction of fluorine by UPW from nine out of 11 processed samples raises concerns about the leaching potential of polymers at the size of microplastics.

To fully examine the extent of potential PFAS present within the polymers, additional investigations are needed to accurately discriminate between inorganic fluorine, organically bound fluorine and PFAS. The SOP was developed to allow easy adaptations and additions to the sample preparation such as an implementation of SPE to separate organically bound fluorine and inorganic fluorine. Determining the extractable fluorine content and aqueous fluorine leaching for polymers is relevant as (i) it enables manufacturers to test their polymer products for leaching during a specific use, (ii) it enables the screening of polymer materials for compliance with potential future regulations, and (iii) it enables product developers to advance the development of polymer products towards materials safe and sustainable by design.

## Supplementary Information

Below is the link to the electronic supplementary material.

Supplementary file1 (DOCX 2.49 MB)

## Data Availability

All data will be made available upon request to the corresponding authors.
