# Supplementary material for: Development of a sample preparation protocol for fast fluorine screening of sealing materials—Considering potential PFAS regulations
Source: Anal Bioanal Chem. 2026 Jun 12;418(15):4825–33. doi: 10.1007/s00216-026-06575-2 (PMC13388568; doi:10.1007/s00216-026-06575-2)
Supplement: Supplementary file 1 — Supplementary file1 (DOCX 2.49 MB) [file 216_2026_6575_MOESM1_ESM.docx]

**Development of a Sample Preparation Protocol for Fast Fluorine Screening of Sealing Materials – Considering Potential PFAS Regulations**

Sebastian Kampf^1‡^, Ronya Mona Wallis^2,1‡^, Lennart Gehrenkemper^1*^, Björn Meermann^1*^

^1^Federal Institute for Materials Research and Testing (BAM), Division 1.1 – Inorganic Trace Analysis (ITA_Lab_), 12489 Berlin, Germany

^2^German Environment Agency (UBA), FG II 2.5 “Laboratory for water analytics”, 12099 Berlin, Germany

^‡^Sebastian Kampf and Ronya Mona Wallis contribute equally as first author to this manuscript

^*^Corresponding authors: Lennart Gehrenkemper and Priv.-Doz. Dr. habil. Björn Meermann

**Supporting Information**

Figures of samples analysed during this study:

| 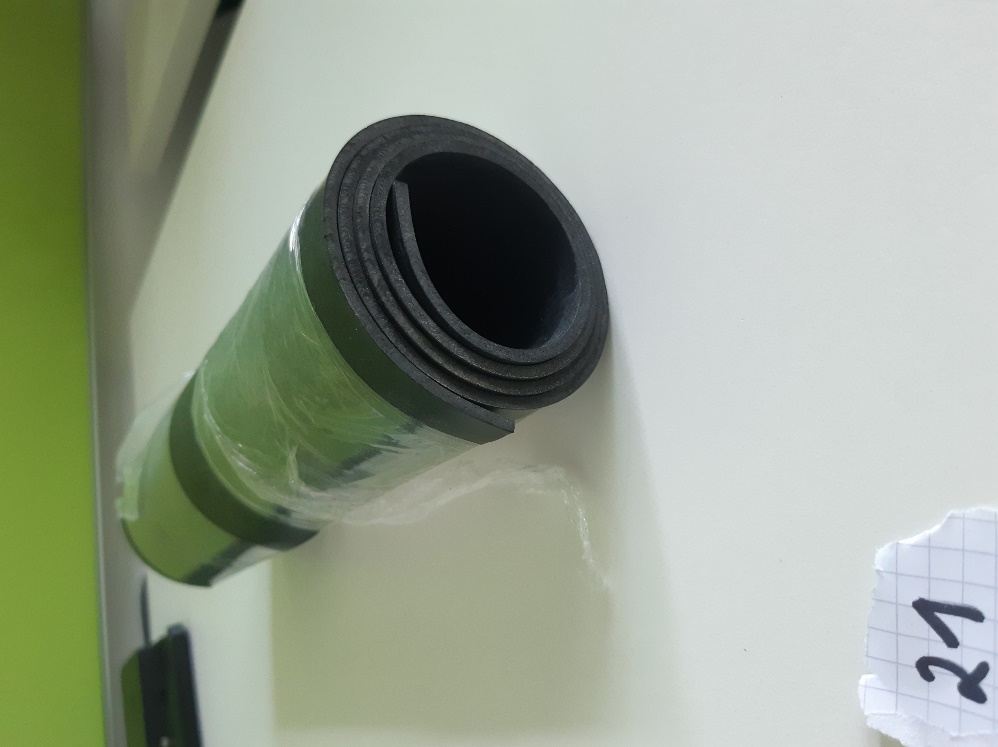 | 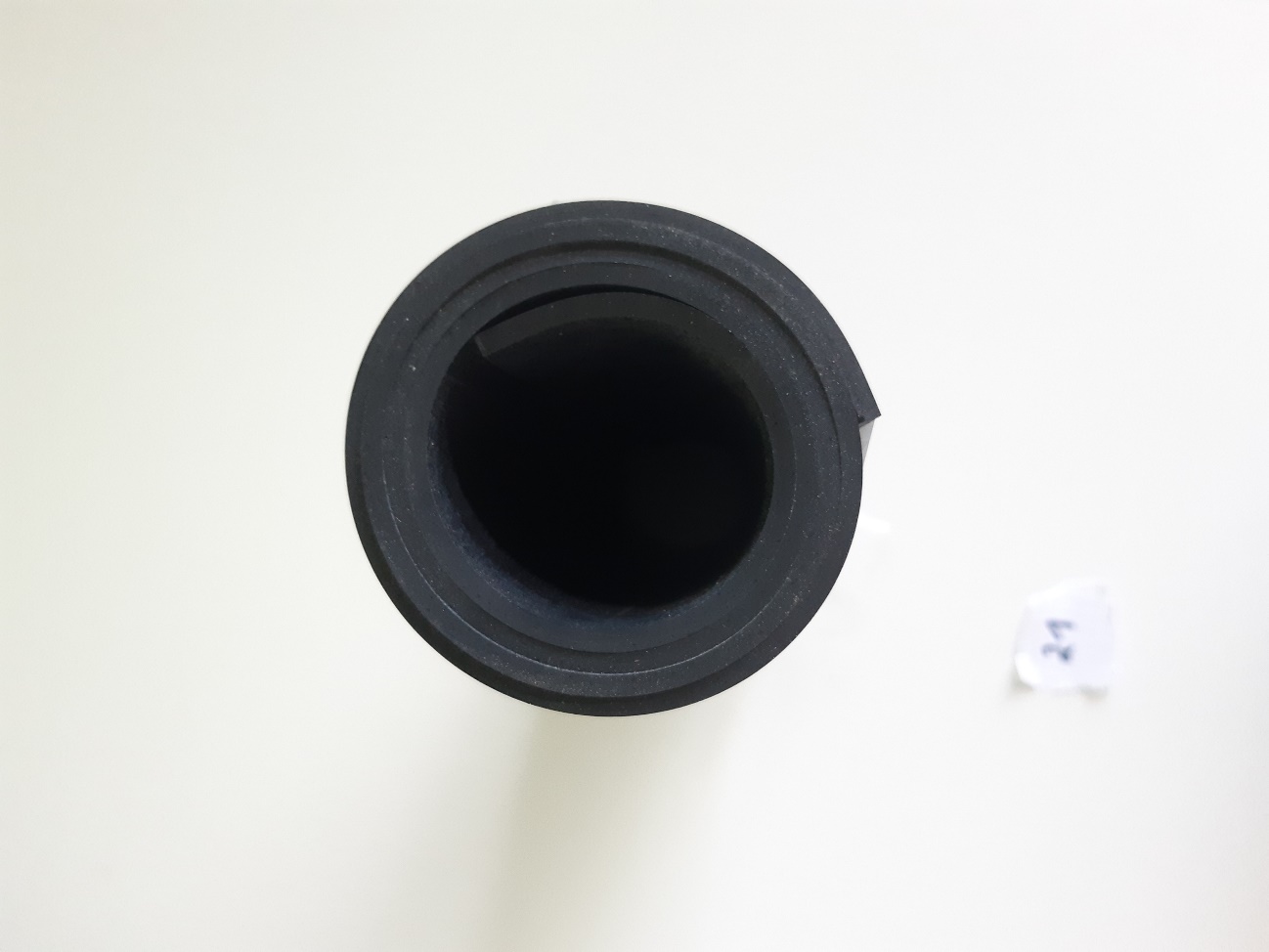 |
| --- | --- |
| Figure 1: Photos of intact sample EPDM 1. |  |
| 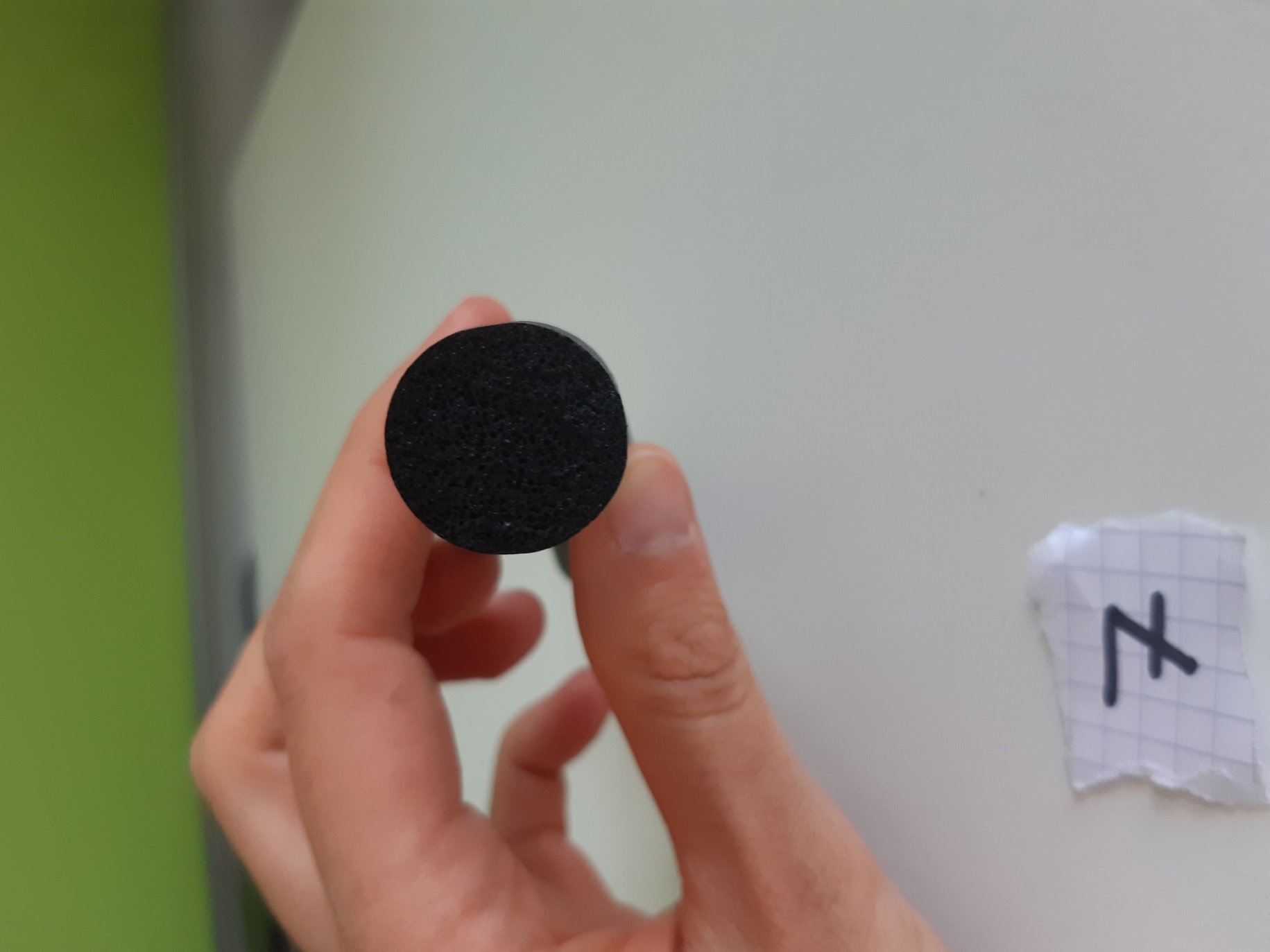 | 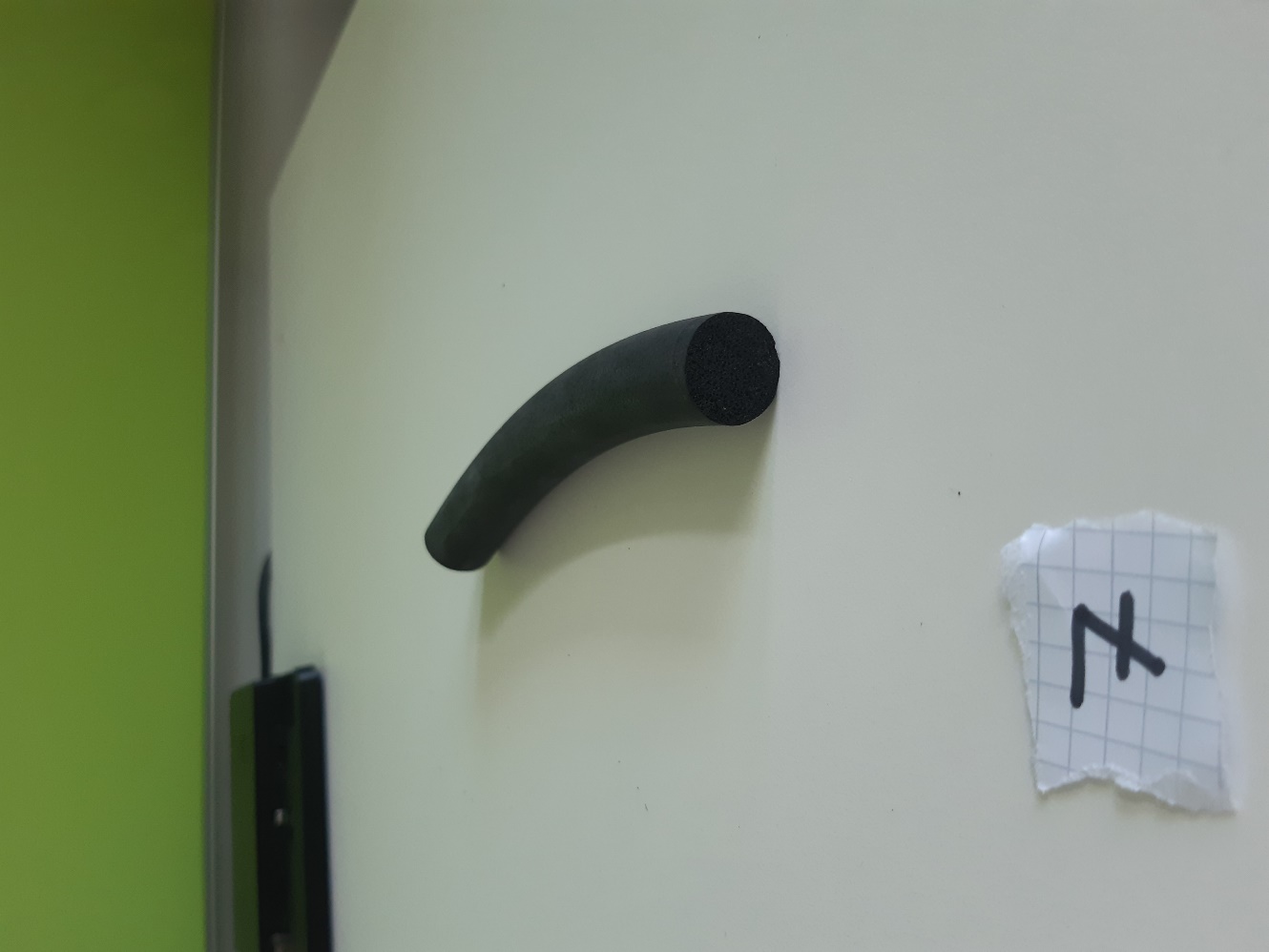 |
| Figure 2: Photos of intact sample EPDM 2. |  |
| 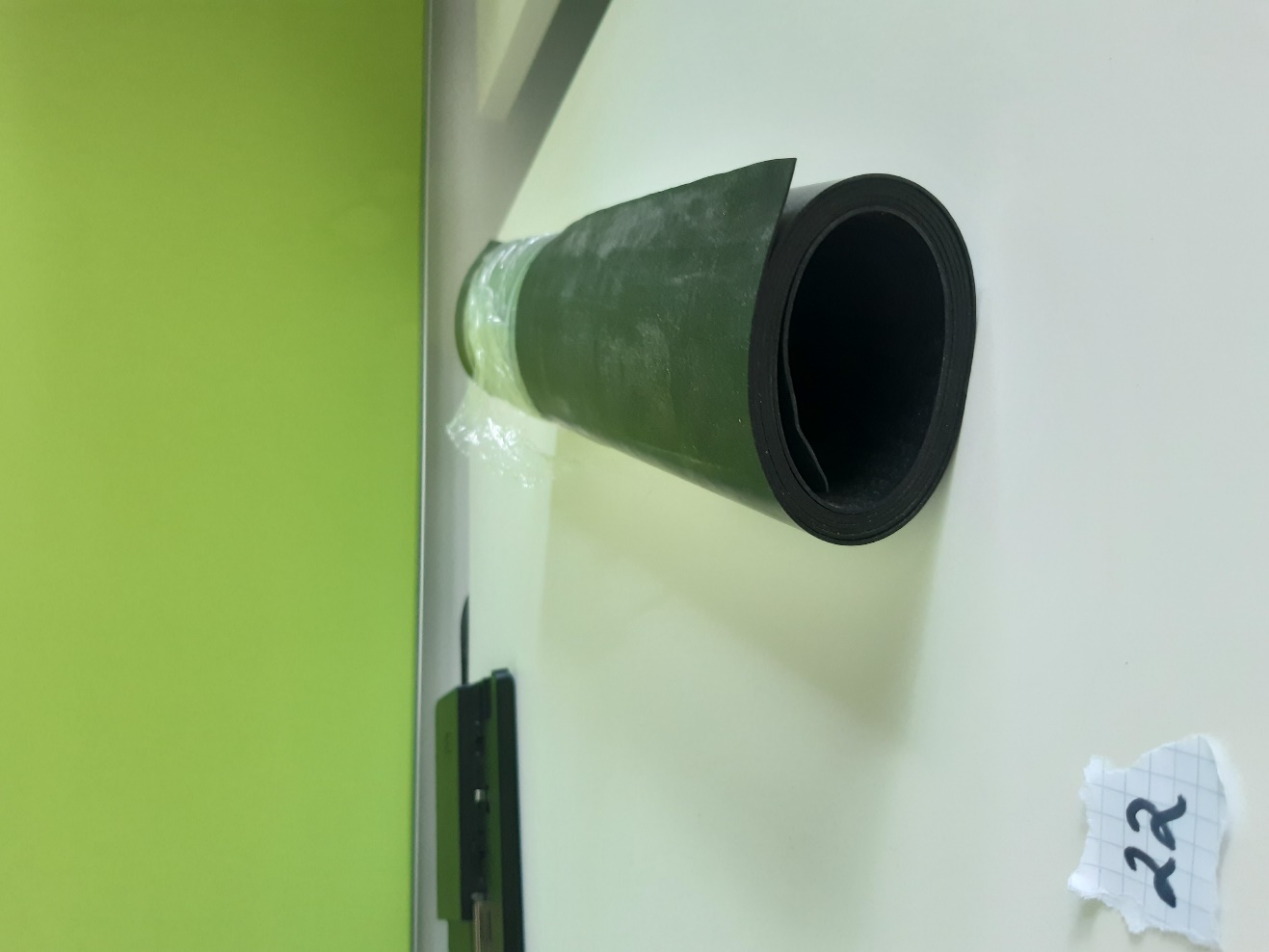 | 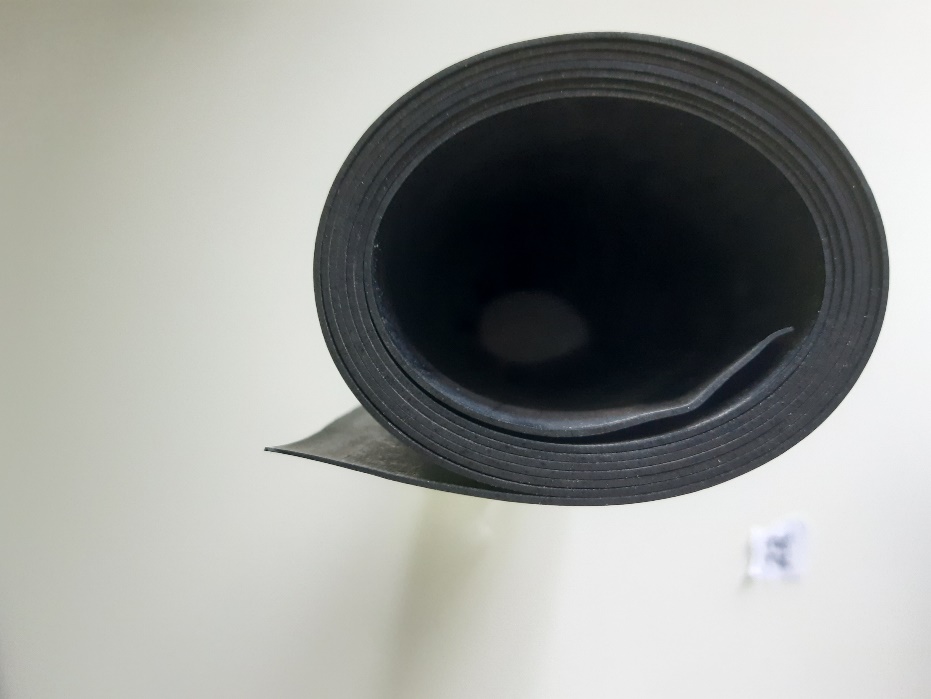 |
| Figure 3: Photos of intact sample SBR 1. |  |
| 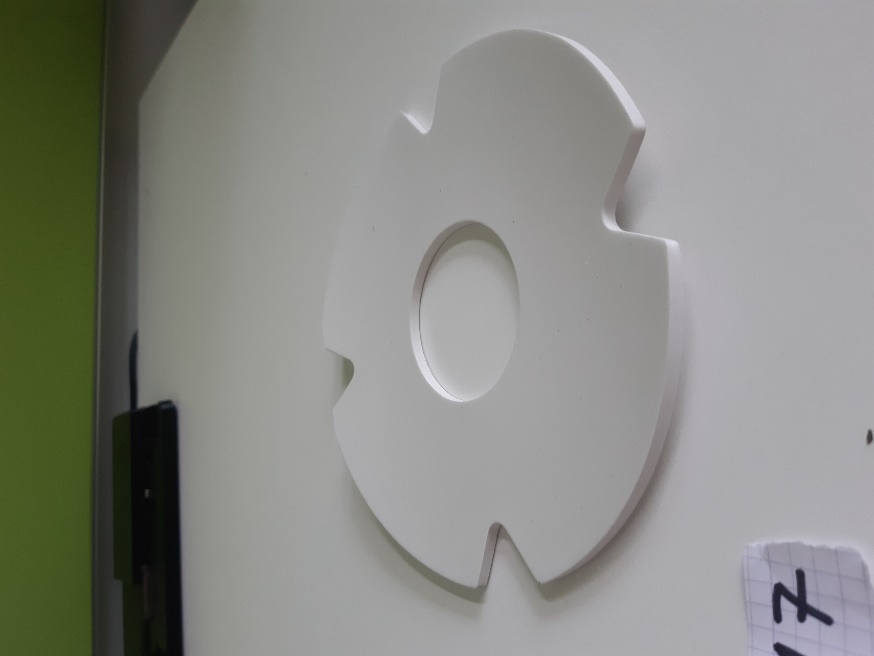 | 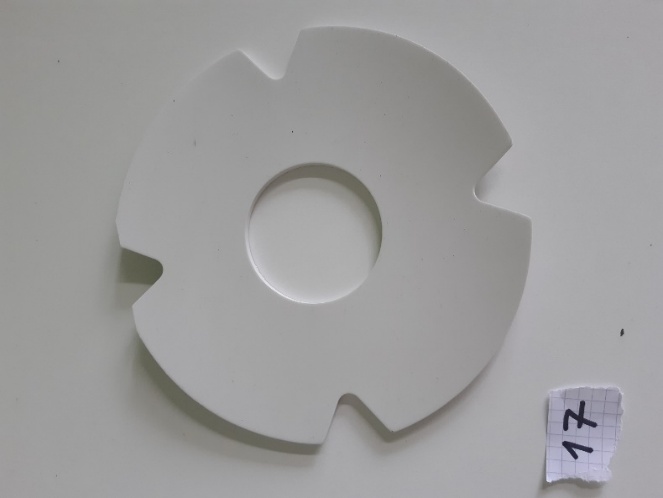 |
| Figure 4: Photos of intact sample SBR 2. |  |
| 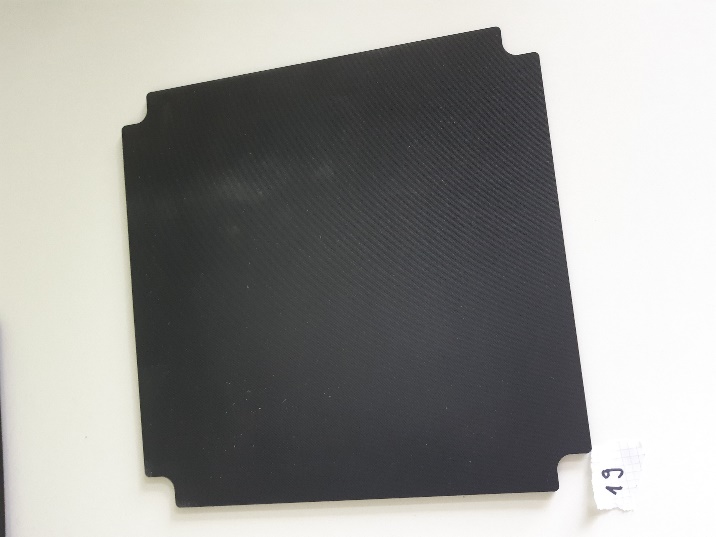 | 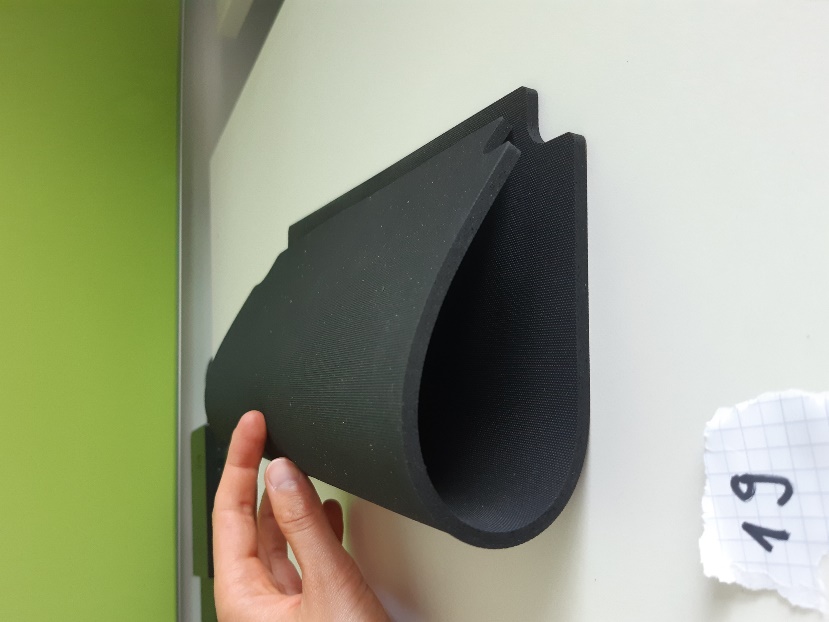 |
| Figure 5: Photos of intact sample CR 1. |  |
| 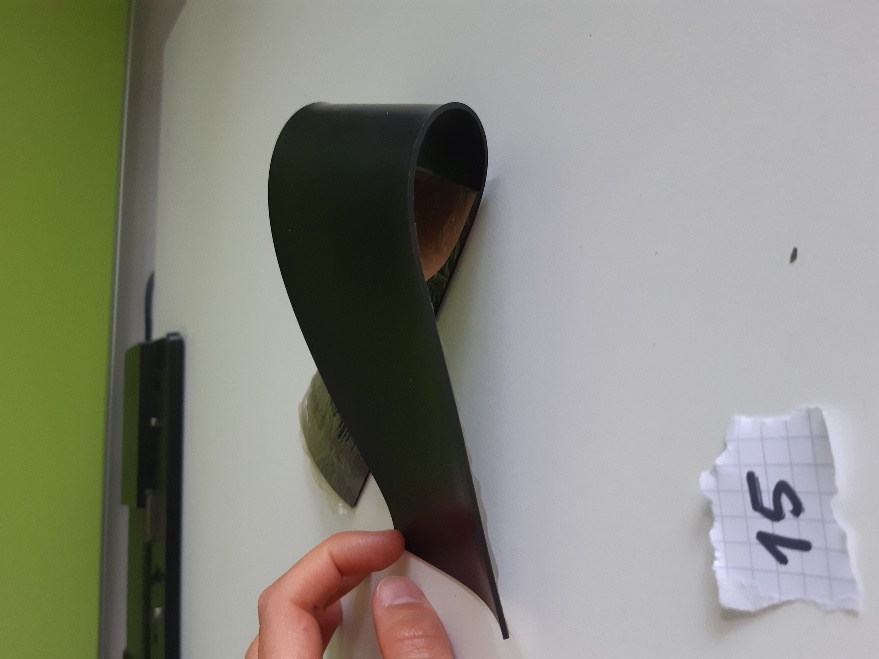 | 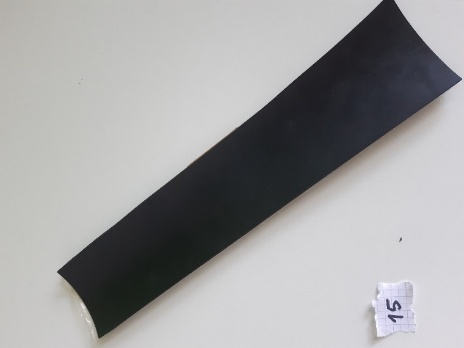 |
| Figure 6: Photos of intact sample CR 2. |  |
| 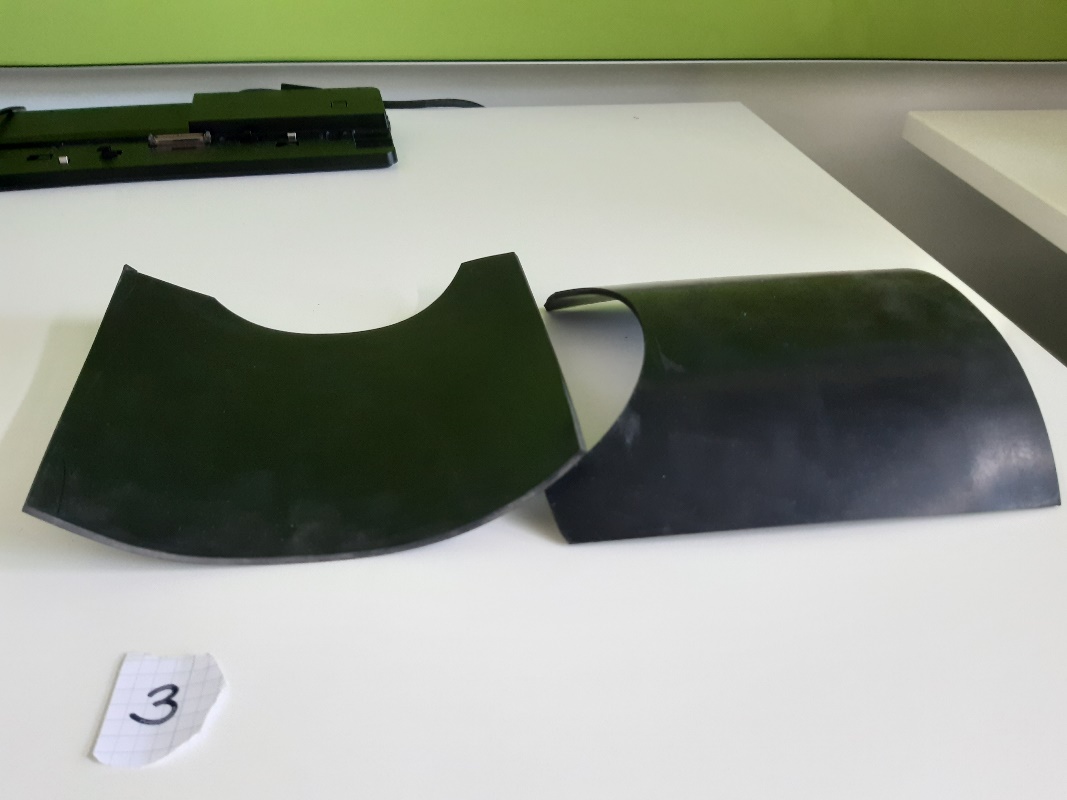 | 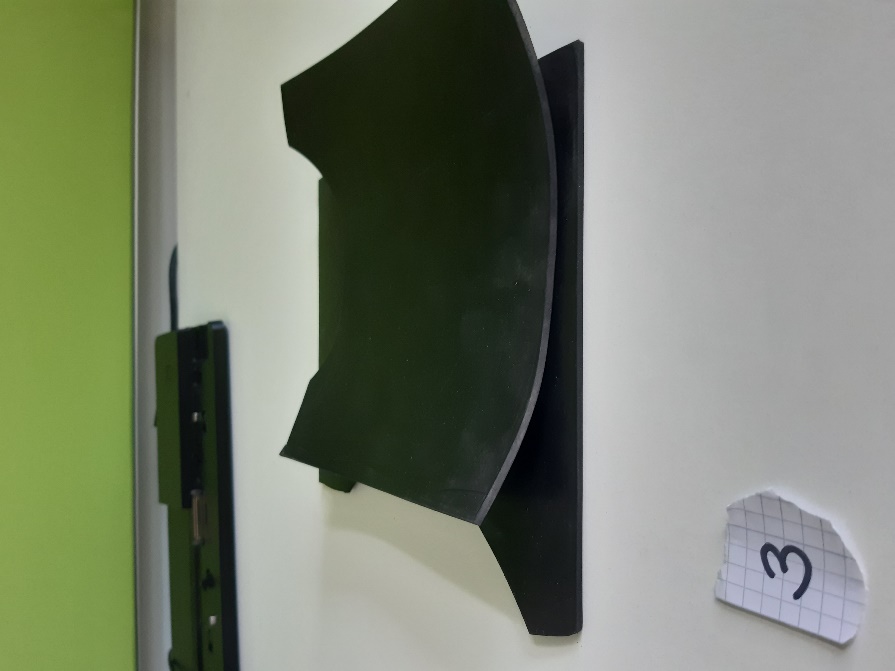 |
| Figure 7: Photos of intact sample NBR 1. |  |
| 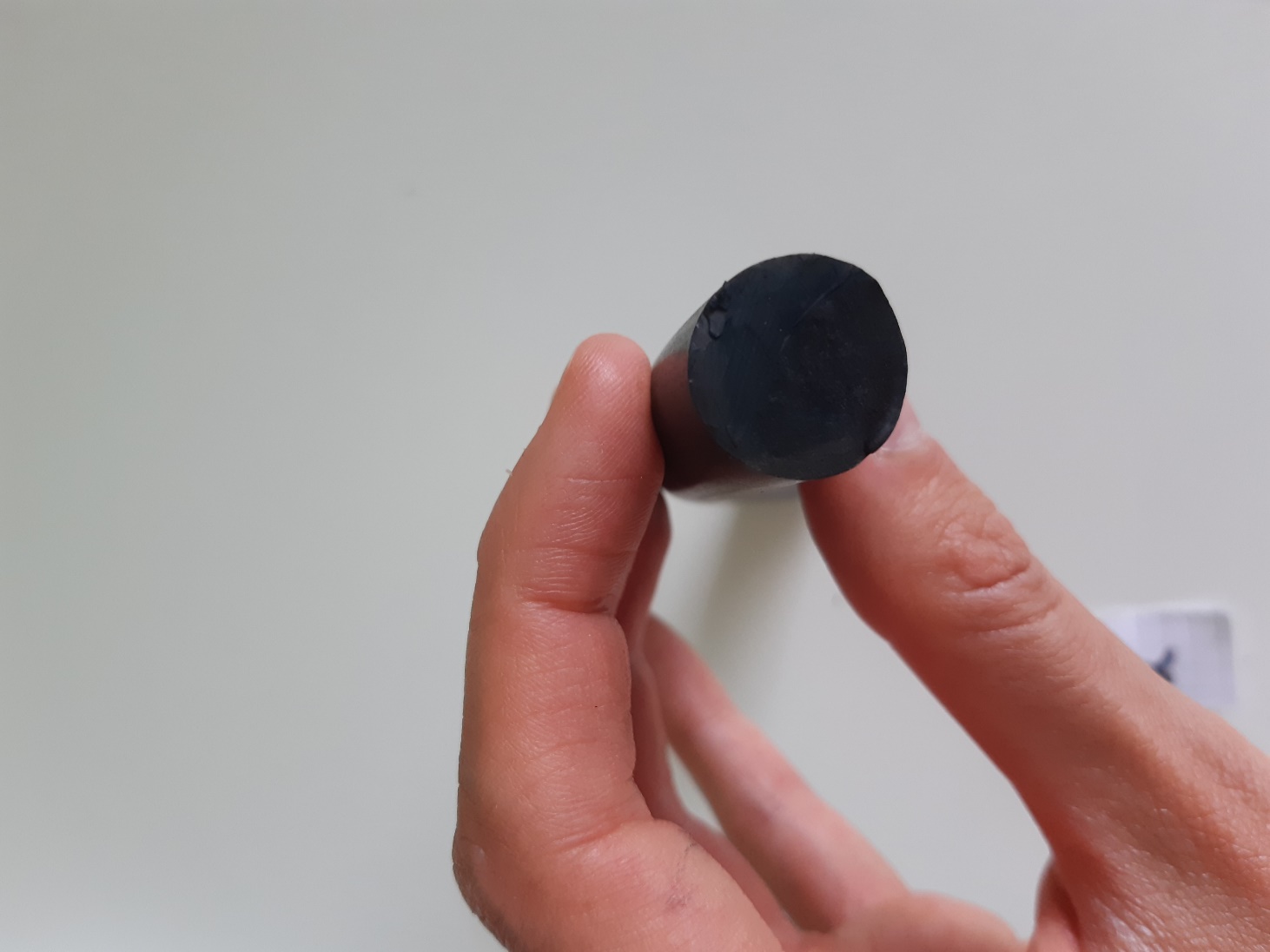 | 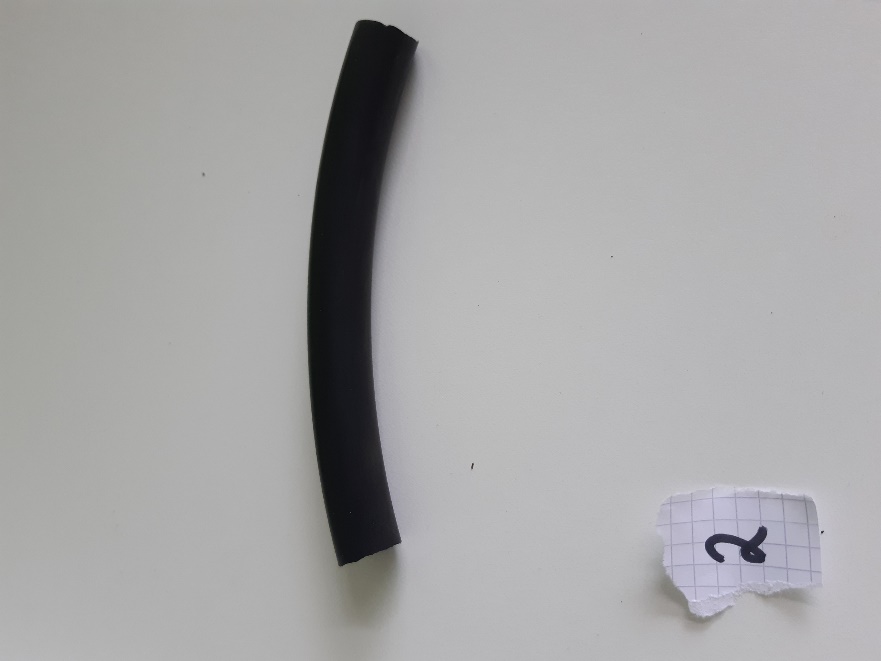 |
| Figure 8: Photos of intact sample NBR 2. |  |
| 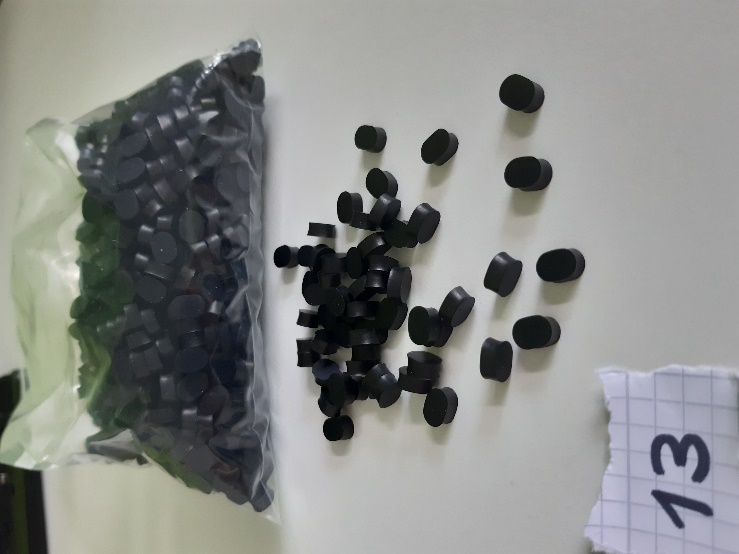 | 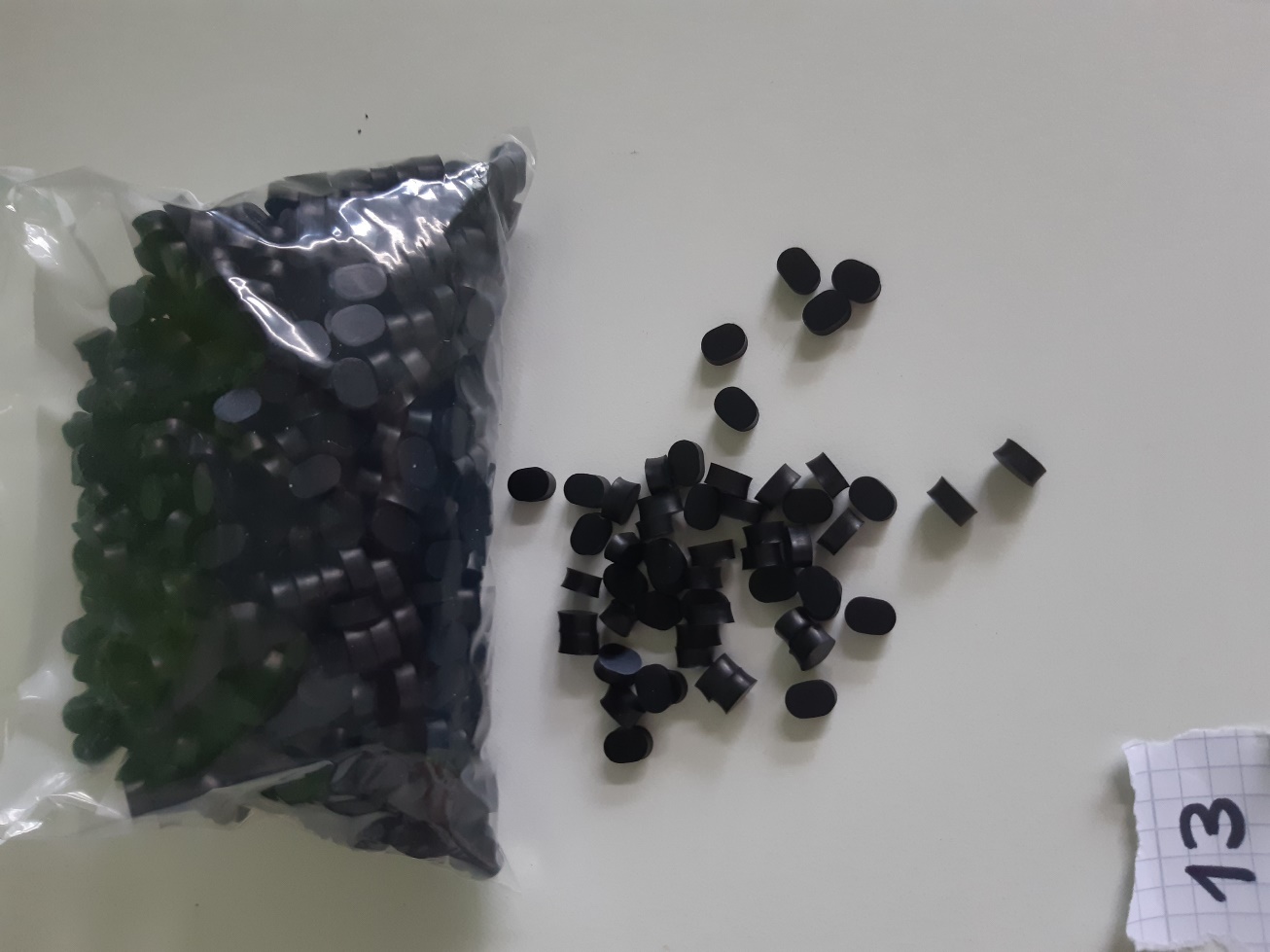 |
| Figure 9: Photos of intact sample IIR. |  |
| 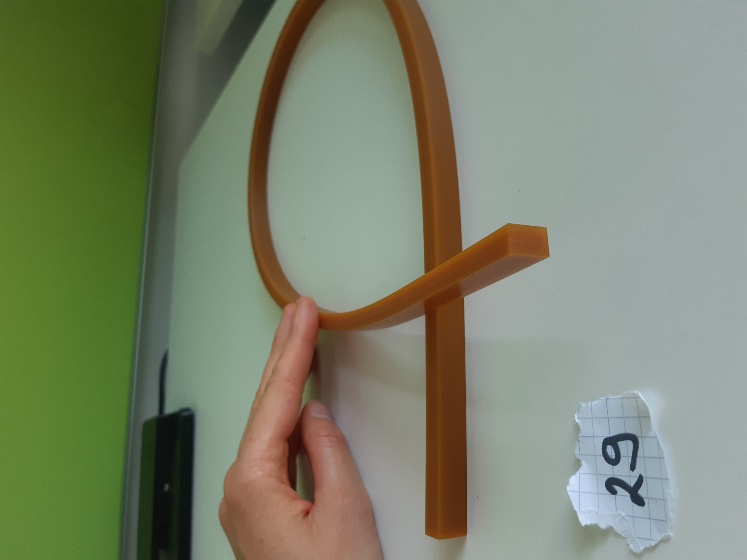 | 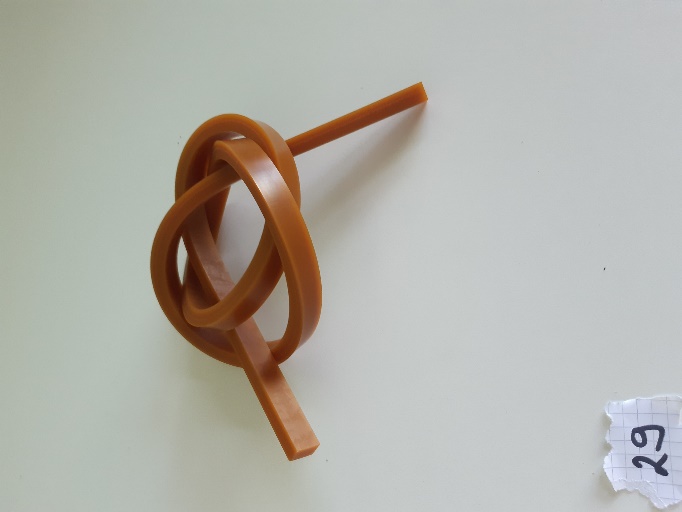 |
| Figure 10: Photos of intact sample PRU. |  |
| 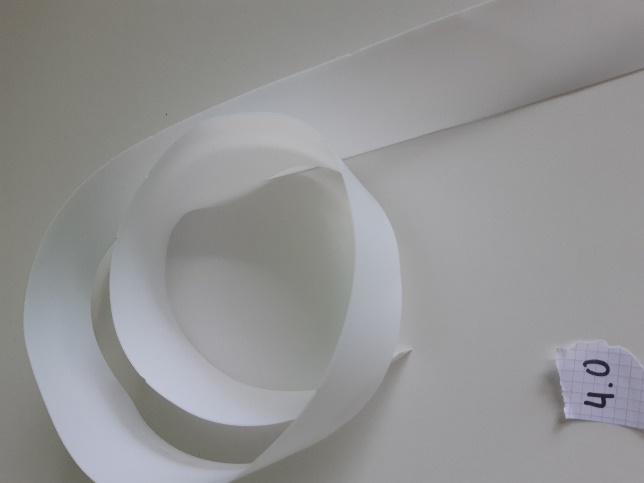 | 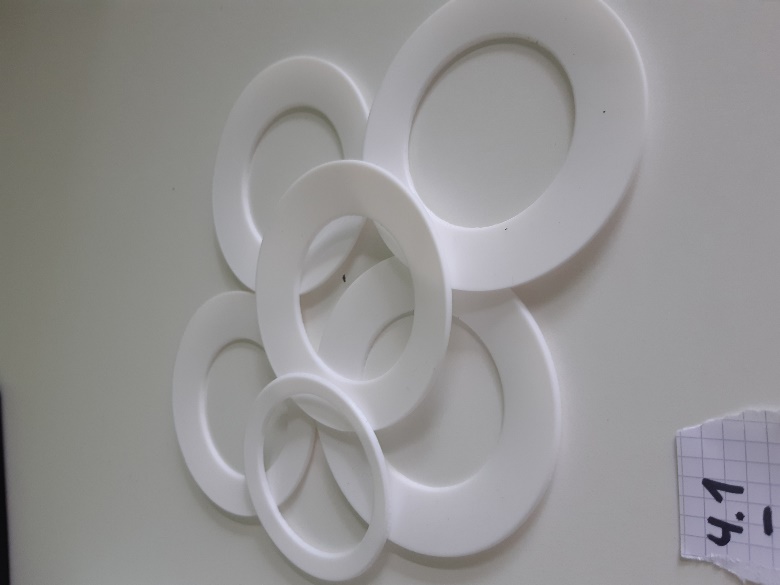 |
| Figure 11: Photos of intact sample PTFE. |  |
| 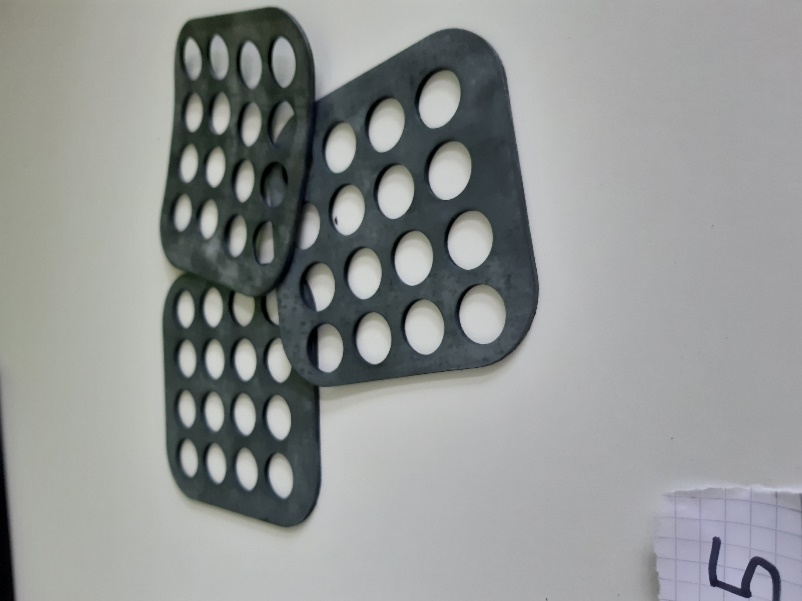 | 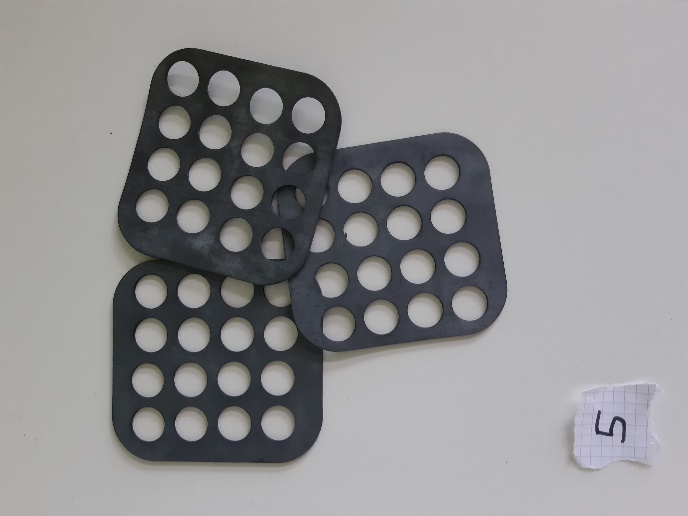 |
| Figure 12: Photos of intact sample FKM. |  |

| Table 1. Injected volumes of sample and modifiers for HR‑CS‑GFMAS measurements [1] | |
| --- | --- |
| Solution | Volume [µL] |
| Ultra-pure water  Sample  Gallium solution (1 g/L)  Sodium acetate solution (10 g/L)  Modifier mix (see above)  Ultra-pure Water | 2  16  9  3  3  2 |

| Table 2: Temperature program during fluorine analysis *via* HR-CS-GFMAS [1] | | | |
| --- | --- | --- | --- |
| Heating Step | Temperature [°C] | Heating Rate [°C/s] | Hold [s] |
| Drying  Drying  Drying  Pyrolysis  Gas Adaptation  Molecule Formation  Clean Out | 80  90  110  500  500  1,550  2,450 | 5  5  5  500  0  1,500  500 | 25  30  20  10  5  6  5 |

| Table 3: Size fractions, obtained masses and mass-fraction of the initial quantity of the investigated polymer samples. | | | | | |
| --- | --- | --- | --- | --- | --- |
| Sample | Size Fraction | Mass [g]  (Mass-fraction of initial quantity) | Sample | Size Fraction | Mass [g]  (Mass-fraction of initial quantity) |
| SBR 1 | 250 µm < x < 500 µm | 14.0 (24 %) | SBR 2 | 250 µm < x < 500 µm | 6.7 (11 %) |
|  | 125 µm < x < 250 µm | 28.9 (50 %) |  | 125 µm < x < 250 µm | 18.9 (30 %) |
|  | x < 125 µm | 4.3 (8 %) |  | x < 125 µm | 6.9 (11 %) |
|  | Loss | 10.0 (18 %) |  | Loss | 29.8 (48 %) |
| EPDM 1 | 250 µm < x < 500 µm | 9.3 (21 %) | EPDM 2 | 250 µm < x < 500 µm | 7.4 (26 %) |
|  | 125 µm < x < 250 µm | 18.1 (40 %) |  | 125 µm < x < 250 µm | 10.2 (36 %) |
|  | x < 125 µm | 2.7 (6 %) |  | x < 125 µm | 4.9 (17 %) |
|  | Loss | 15.0 (33 %) |  | Loss | 6.0 (21 %) |
| CR 1 | 250 µm < x < 500 µm | 6.7 (11 %) | CR 2 | 250 µm < x < 500 µm | 1.5 (4 %) |
|  | 125 µm < x < 250 µm | 25.6 (41 %) |  | 125 µm < x < 250 µm | 19.4 (53 %) |
|  | x < 125 µm | 8.3 (13 %) |  | x < 125 µm | 6.6 (18 %) |
|  | Loss | 22.3 (35 %) |  | Loss | 9.4 (25 %) |
| NBR 1 | 250 µm < x < 500 µm | 5.8 (9 %) | NBR 2 | 250 µm < x < 500 µm | 3.2 (7 %) |
|  | 125 µm < x < 250 µm | 24.4 (40 %) |  | 125 µm < x < 250 µm | 21.1 (45 %) |
|  | x < 125 µm | 14.7 (24 %) |  | x < 125 µm | 8.6 (19 %) |
|  | Loss | 16.8 (27 %) |  | Loss | 13.7 (29 %) |
| PU | 250 µm < x < 500 µm | 18.3 (34 %) | IIR | 250 µm < x < 500 µm | 2.6 (4 %) |
|  | 125 µm < x < 250 µm | 17.2 (32 %) |  | 125 µm < x < 250 µm | 17.6 (26 %) |
|  | x < 125 µm | 3.3 (6 %) |  | x < 125 µm | 23.2 (35 %) |
|  | Loss | 14.9 (28 %) |  | Loss | 23.6 (35 %) |
| PTFE | 250 µm < x < 500 µm | Not Measured |  |  |  |
|  | 125 µm < x < 250 µm | Not Measured |  |  |  |
|  | x < 125 µm | Not Measured |  |  |  |
|  | Loss | Not Measured |  |  |  |

References

[1] Metzger, M., et al., *Screening method for extractable organically bound fluorine (EOF) in river water samples by means of high-resolution–continuum source graphite furnace molecular absorption spectrometry (HR-CS GF MAS).* Analytical and Bioanalytical Chemistry, 2019. **411**(19): p. 4647–4660.
